# Impact of COVID-19 Pandemic on Patients with Immune Thrombocytopaenia

**DOI:** 10.3390/medicina57030219

**Published:** 2021-03-01

**Authors:** María-Teresa Álvarez Román, Víctor Jiménez Yuste, Sara García Barcenilla, Andrés Ramírez López, Elena Monzón Manzano, Beatriz de la Cruz Benito, Paula Acuña Butta, María Isabel Rivas Pollmar, Roberto Trelles Martínez, Elena González Zorrilla, Mónica Martín Salces, Tamara Cebanu, Nora V. Butta

**Affiliations:** 1 Hematology Unit, La Paz University Hospital-IdiPAZ, Paseo de la Castellana 261, Hospital Universitario La Paz, 28046 Madrid, Spain; vjyuste@gmail.com (V.J.Y.); ehemostasia@gmail.com (S.G.B.); andres.ramirez1793@gmail.com (A.R.L.); elenamonzonmanzano@hotmail.com (E.M.M.); bdelacruzbenito@gmail.com (B.d.l.C.B.); paulaacbu@gmail.com (P.A.B.); mirivas718@gmail.com (M.I.R.P.); rotrelles@gmail.com (R.T.M.); elenaehemostasia@gmail.com (E.G.Z.); monicamsalces@gmail.com (M.M.S.); tamara.ce@hotmail.es (T.C.); nora.butta@salud.madrid.org (N.V.B.); 2 Medicine Department, Autonomous University of Madrid, Arzobispo Morcillo 4, 28029 Madrid, Spain

**Keywords:** COVID-19, immune thrombocytopaenia, vulnerable population, telemedicine

## Abstract

*Background and Objectives*: The aim of this study was to determine the impact of the COVID-19 pandemic on the lives of patients with immune thrombocytopaenia (ITP) treated at our hospital. *Materials and Methods*: The study was conducted in the Community of Madrid, which has the highest number of COVID-19 cases in Spain. We included 143 adult patients with ITP (130 with chronic ITP, 8 with persistent ITP, and 5 with newly diagnosed ITP). We conducted a telephone survey to collect the data and created a registry. *Materials and Methods*: Overall, 24 patients presented symptoms suggestive of COVID-19, which was confirmed by RT-PCR in 8 cases. The cumulative incidence of confirmed SARS-CoV-2 infection was higher in the patients with ITP than in the Madrid population. There were no differences in the disease incidence or clinical course of infection in the patients treated with immunosuppressants. Almost all of the patients reported adherence to the prescribed treatment, although 49.2% of the hospital visits were either cancelled or postponed, 17.2% because of the patients’ fear of coming to the centre. Nearly half of the cohort was considered vulnerable, and 17% had been granted a dependency or disability benefit. *Conclusions*: COVID-19 had a major impact on the psychosocial, occupational, and quality of care of patients with ITP.

## 1. Introduction

On 30 January 2020, the World Health Organisation declared the coronavirus disease 2019 (COVID-19) outbreak to be a public health emergency of international concern, and, on 1 February 2020, the organisation characterised COVID-19 as a pandemic.

Spain has been one of the most affected countries, with thousands of cases, especially in the Madrid region. La Paz University Hospital admitted over 3000 patients with COVID-19, one of the largest single-site cohorts in Europe [1,2,3].

At the beginning of the pandemic, our haematology department established protocols to minimise the risk of transmission. This department treats a cohort of adult patients with immune thrombocytopaenia (ITP), who, when treated with immunosuppressants (such as corticosteroids and rituximab), might be at greater risk of developing a serious acute respiratory syndrome-coronavirus-2 (SARS-CoV-2) infection. We therefore established a telemedicine program to monitor these patients, scheduling remote visits to keep them out of the hospital [4].

The involvement of platelets in COVID-19 severity is a matter of debate. On one hand, platelet counts do not seem to differ between patients with severe disease and those exhibiting mild disease [5]; however, platelets are hyperactivated in severe COVID-19 [6] and are involved in the thrombotic complications observed in these patients [7].

The Spanish government’s strategy to reduce the spread of the pandemic was a lockdown and social distancing. The aim of our study was to determine how these changes affected the lives of patients with ITP treated in our unit. To this end, we interviewed the patients regarding the support they had required from the hospital and the ways in which it had been delivered. We aimed to determine how the infection was managed, assessing the treatment adherence, and identifying the factors influencing the potential loss of adherence. We also aimed to assess the impact of the lockdown on the musculoskeletal system and gather the psychosocial aspects of the lockdown, such as the need for family and social support, the employment situation, and the dependency/disability benefit granted by the government. Aspects regarding the quality of care were also assessed.

## 2. Materials and Methods

Since 16 March 2020 and following the protocol we developed due to the pandemic, we encouraged patients not to come to the hospital except in case of emergency. For any other situation, we established a telemedicine program to replace on-site visits. To detect whether patients with ITP had COVID-19, we contacted our entire patient cohort by phone to evaluate their medical and psychosocial status. This process was a crucial aspect of our study because had the patients not needed hospitalisation or care from our emergency department, we might have had no knowledge of their SARS-CoV-2 infection.

Due to the high burden of the pandemic on our national health system, we could not perform polymerase chain reaction (PCR) confirmation of the COVID-19 diagnoses on all suspected cases. Therefore, patients who presented respiratory symptoms were classified as possible (mild acute respiratory infection for which no microbiological diagnostic test had been performed) or confirmed (meeting laboratory confirmation criteria by PCR) COVID-19 cases, according to the Ministry of Health’s Procedure for Action against Cases of Infection with the New Coronavirus SARS-CoV-2 [8].

The project was approved by the Ethics Committee of La Paz University Hospital (ethics code PI-4141, 4 May 2020). Patient data were collected from 5 May 2020 to 30 May 2020.

Patients were invited to participate by phone after they were explained the aims of the study, and their oral consent was recorded in their medical history. Those who agreed to participate responded to a questionnaire we developed for the study, the variables of which are shown in the Supplementary File.

The data were treated confidentially in accordance with Regulation (EU) 2016/679 of the European Parliament and of the Council of 27 April 2016 on Data Protection and the Organic Law 3/2018, of 5 December, Protection of Personal Data and Guarantee of Digital Rights.

The statistical analysis was performed by the Statistical Unit of La Paz University Hospital. For the data analysis, the patients were stratified according to ITP type, ITP medication, and patient age, and the qualitative population variables are expressed as absolute frequencies and percentages. The cumulative incidence was estimated with a 95% confidence interval.

## 3. Results

### 3.1. Characteristics of Patients with Immune Thrombocytopaenia

This registry included 147 patients diagnosed with ITP who were contacted by phone. Of these, 134 had chronic ITP, 8 had persistent ITP, and 5 were newly diagnosed, classified according to Rodeghiero et al. [9]. Of the five patients with newly diagnosed ITP, two were associated with COVID-19 [10]. The cohort’s mean age was 59.38 (20–92) years old.

Table 1 shows that, at the time of the evaluation, only 47 patients with ITP were undergoing therapy for the disease, whereas the other 96 were not being treated but had required treatment in the past three months. A total of 34 patients with ITP were being treated with thrombopoietin receptor agonists (TPO-RA), 4 with corticoids, 2 with immune globulin intravenous (IGIV), and 7 with combined schemes (corticoids and IGIV). A total of 22 patients who were not being treated and 5 who were undergoing TPO-RA therapy had undergone a splenectomy. Moreover, 32 patients with ITP were undergoing antithrombotic or antiplatelet therapies or both due to various conditions. The main reason for anticoagulant therapy was atrial fibrillation (seven patients), followed by a prosthetic valve (three patients), thromboembolic disease (two patients), and antiphospholipid syndrome (one patient). Antiplatelet therapy was prescribed to six patients for ischemic cardiopathology, to five for vascular disease, to three for antiphospholipid syndrome, and to one patient each due for congenital cardiopathy, thrombocytosis secondary to splenectomy, and ischemic myelitis.

### 3.2. Management of the Disease by the Health Care and Community Support System

In terms of patient-reported musculoskeletal pain during the lockdown, 85 answered that they had no pain. Of the 58 patients reporting pain of any kind, 22 reported acute pain, and 36 reported chronic pain. In addition, 37 patients controlled the pain pharmacologically, 19 controlled it without pharmacological products, and 2 patients did not answer this question.

Of the 47 patients undergoing treatment for ITP, 46 were treatment-adherent during the pandemic, whereas 1 was identified as partially adherent by a haematologist.

A total of 59 patients reported that they had adapted their physical activity to the lockdown, whereas 82 had not, and 2 did not answer the question. Among the 140 patients who responded to how they felt regarding the lockdown, 52 indicated that they had observed a stricter lockdown than others due to ITP.

Due to the COVID-19 pandemic, numerous appointments were postponed or cancelled. Of the appointments scheduled between March 9th and 28th May 2020 (*n* = 59), 6.8% were cancelled, and 42.4% were postponed, and 30.5% of the patients were seen in person, and 20.3% did not know/remember if they had had an appointment to go to the hospital. Some 17.2% of the appointments that were cancelled or delayed were due to the patient’s fear of coming to the centre.

### 3.3. Psychosocial Aspects

A total of 117 (81.8%) patients spent the lockdown accompanied by their families, 4 (2.8%) in a residence, 5 (3.5%) with friends, and 17 (11.9%) alone.

We were able to collect data on patient vulnerability in a socio-familiar context for 141 of the patients: 75 (53.2%) were categorised as nonvulnerable, while 66 (46.8%) were categorised as vulnerable; 24 (17%) had been granted a dependency or disability benefit, whereas 117 (83%) had no benefits or assistance in this area. Overall, 12 of the patients sought help from their family and this requirement was fulfilled by the social services of the Autonomous Community of Madrid in 83.3% of the cases.

Furthermore, 20 patients were declared dependent for daily activities, and 122 were recognised as independent to perform daily activities.

We were able to collect occupational-related data for 139 patients. The distribution was 64 (45%) pensioners, 46 (33%) full/part-time workers, 12 (8.6%) individuals under the Temporary Employment Regulation Action (expediente de regulación temporal de empleo (ERTE)), 10 (7.2%) homemakers, and 5 (3.6%) individuals on medical leave.

### 3.4. SARS-CoV-2 Infection in Patients with Immune Thrombocytopaenia

A total of 24 patients were defined as probable or confirmed cases of coronavirus infection. Eight of these were confirmed by PCR, and 16 had suggestive signs and symptoms of coronavirus infection.

Regarding the analysis of the cumulative incidence of COVID-19 in the patients with ITP, the closer follow-up of these patients, when compared with that of the general population of Madrid [11], can be considered a bias in the data collection. There were no significant differences in the proportion of patients with COVID-19 between persistent ITP and chronic ITP. Table 2 shows the features of the patients with and without suspected COVID-19. We employed a chi-squared test to correlate several variables but found no significant differences between the groups. Similarly, the progression of COVID-19 was analysed in the patients with ITP treated with anticoagulants or antiplatelets. There were no differences in the haemorrhagic or thrombotic complications among the patients who did not undergo these therapies.

## 4. Discussion

Although patients with ITP infected with SARS-CoV-2 and the development of ITP in the context of COVID-19 have been reported [12,13], this is the first study to evaluate how the COVID-19 pandemic has affected patients with ITP treated at the haematology unit of a high-complexity hospital centre of the Community of Madrid.

The objective of the study was to determine the incidence of COVID-19, the disease progression if it occurred, and the impact on these patients’ psychosocial aspects and quality of care.

We cannot conclude that the cumulative incidence of COVID-19 in our series was relatively higher than that observed in the Community of Madrid for the same period because the closer communication with the patients in our unit gave us the opportunity o perform more tests and detect more cases. One of the limitations of this study is the lack of PCR confirmation of the COVID-19 in the patients with suspicious symptoms, the result of the pandemic, and the burden it placed on Spain’s national health system, which was unable to fulfil all diagnostic necessities.

We might assume that patients with ITP treated with corticosteroids or other immunosuppressants would have greater vulnerability to SARs-COV-2 infection. Nevertheless, the few cases of patients with ITP treated with immunosuppressants in our series did not allow us to draw a conclusion on this aspect. None of the splenectomised patients who developed COVID-19 had a bacterial infection or underwent prophylactic antibiotic treatment, although we did ensure that their vaccinations were updated.

Only three of the patients who developed ITP after COVID-19 required hospital admission; the rest were managed on an outpatient basis, despite the fact that 44.8% had risk factors (high blood pressure, diabetes mellitus, chronic obstructive pulmonary disease) for developing severe COVID-19 disease. Several published studies have reported that SARS-CoV-2, unlike infection by other viruses (such as adenovirus, rhinovirus, norovirus, influenza, and respiratory syncytial virus) does not cause more disease in immunosuppressed patients [14,15].

Habernan et al. recently reported that patients with other immune-mediated inflammatory diseases (such as rheumatoid arthritis and inflammatory bowel disease) undergoing immunomodulatory treatment are not at increased risk of complications, possibly because these immunomodulatory agents control the inflammatory response produced by SARS-CoV-2 [16]. Nevertheless, this issue is controversial. Kow and Hasan [17] discussed the variety of clinical characteristics and outcomes of patients with various autoimmune diseases (rheumatoid arthritis, psoriasis) and concluded that the differences might depend on the target of the immunomodulatory treatment. In contrast, rituximab might impair the priming of antibody responses to neutralise viral replication, inducing an unfavourable clinical outcome, biologic cytokine inhibitors might diminish the cytokine storm associated with COVID-19, thereby producing less severe disease. Moreover, the participation of B cells in COVID-19 outcomes appears to extend beyond their ability to produce antibodies, given that differences in the progression of this disease were found between patients who lacked B cells (patients with agammaglobulinemia) and patients with dysfunctional B cells (patients with primary antibody deficiencies). Patients with dysfunctional B cells present a severe form of the disease because their B cells release IL-6, which increase the inflammation level [18].

We evaluated the incidence of thrombotic events, given that patients with ITP have an increased incidence of arterial and venous thrombosis, and this risk increases with certain treatments, such as splenectomy and TPO-RA, and even more with COVID-19 infection [15].

None of the 24 patients diagnosed with ITP with suggestive COVID-19 developed thrombotic events during the infection despite the fact that four had been administered thrombopoietin receptor agonists (TPO-RA), six had been splenectomised, and seven had other prothrombotic risk factors. These last seven patients were anticoagulated with acenocoumarol or direct-acting anticoagulants and continued this treatment during the SARS-CoV-2 infection.

None of the patients treated with eltrombopag who had COVID-19 developed hepatotoxicity. We can therefore state that COVID-19 infection did not hinder the identification of hepatotoxicity as an adverse effect of eltrombopag in our series, although this is based on a few cases [15].

The psychosocial effects of the COVID-19 pandemic constitute a global challenge for health care. We had to limit all unnecessary activities to mitigate the harmful effects of this pandemic. We therefore cancelled or postponed visits to the hospital and established a telemedicine protocol to attend to these patients. We observed that the pandemic was a demoralising experience that patients faced with fear, sadness, and anxiety due to the possibility of being infected with SARS-CoV-2 and because they felt that the pandemic situation relegated their illness to low priority, with detrimental consequences on their health. These reported feelings are in accordance with the detrimental effects on mental health caused by isolation, given that social networking has been shown to be associated with both physical and psychological well-being [19]. Having a chronic illness appears to increase the probability of suffering anxiety, and older adults show more psychological symptoms compared with their younger counterparts [20]. Our cohort included 130 patients with chronic ITP, and 59.3% of them were older than 65 years old, which explains their distress while in confinement. Similarly, patients with other chronic diseases such as cancer [21], allergic diseases [22], inflammatory bowel disease [23], and haematological malignancies [24] were more likely to report nervousness, anxiety, depression, loneliness, and hopelessness. Being older and having a chronic disease led to a tighter, self-imposed, or family-imposed restriction due to the fear of SARS-CoV-2 infection. The effect of the pandemic on our community has transcended the social and health contexts and has even generated discussion concerning the relationship between religion and health [25].

Overall, 40% of the patients with ITP reported musculoskeletal pain, but there were no significant differences between the patients with and without COVID-19, perhaps because of the low number of patients in the groups. It has been reported that one of the most common symptoms in COVID-19 patients is the triad of myalgia, physical fatigue, and muscle weakness [26]. We cannot rule out the involvement of social interaction restrictions and lockdown in the onset of musculoskeletal pains because these restrictions would affect an individual’s pain threshold [27] and because the lack of physical activity would lead to musculoskeletal pain, especially upper and lower back pain [28]. Another study concluded that socially isolated individuals, regardless of age, have relatively higher risks of impaired self-rated health and musculoskeletal issues [29]. Another issue to consider is that musculoskeletal pain is one of the most common adverse effects of therapies for ITP, such as TPO-RA [30].

Anxiety and mental unrest increased due to the fear of unemployment and financial crises. Unfortunately, this epidemic led to extensive job losses due to the cessation of economic activities; a total of 13.6% of the active patients with ITP (younger than 65 years old) accessed an ERTE, which is a temporary suspension of contracts or a reduction in working hours.

We therefore contacted all patients with ITP who were being treated at our centre to determine their needs. As a result of these telephone calls, we realised that our patient cohort had an increased need to communicate, probably due to the characteristics and duration of the lockdown. Unfortunately, there is no ITP patient association to reduce social isolation in Spain. We also conducted this survey with a cohort of patients with coagulopathies who are followed-up at our centre. Although they were concerned about the pandemic, they were less anxious than the patients with ITP. This difference could be due to the social work performed by the associations of patients with coagulopathies in Spain (FEDHEMO, Spanish Federation of Hemophilia, and ASHEMADRID, Madrid Hemophilia Association), which reduces negative outcomes and improves patient health and quality of life [31].

Taking into account the evolution of the pandemic, the implemented telemedicine protocols should remain in place to guarantee our patients receive good quality care. Nevertheless, we are aware of the risk of not detecting certain side effects of treatments during televisits, which can potentially lead to serious complications or adverse events [32]. There are recommendations for preventing these complications; for example, TPO-RA should be avoided in patients with acute ITP and COVID-19 due to the higher risk of thrombosis and hepatotoxicity [33]. For patients with ITP who are COVID-19 negative, the advice to avoid steroid use to maintain their immune system active against possible SARS-CoV-2 infection [34]. The implementation of a nursing health care service could be useful for the closer follow-up of patients and to obtain blood samples for analysis in hospital laboratories.

Immunisation of the population is just beginning, and outbreaks of coronavirus might occur; protecting older adults is therefore critical. Telemedicine can help, both at the peak of the pandemic and in the coming months, to ensure patients’ continuity of care.

## 5. Conclusions

COVID-19 has had a major impact on the psychosocial, occupational, and quality-of-care elements of patients with ITP, which need to be remedied with recovery plans. It would also be advisable to establish a Spanish society for patients with ITP and their caregivers to empower them through education, advocacy, research, and support.

## Figures and Tables

**Table 1:** Therapeutic treatments of patients with immune thrombocytopaenia.

	Total Cases	Anticoagulant Therapy (*n* = 13)	Antiplatelet Therapy (*n* = 17)	Antiplatelet and Anticoagulant Therapy (*n* = 3)	Splenectomy (>5 Years)
None		6	13	1	22
	96	Acenocoumarol: 2 Apixaban: 1 Rivaroxaban: 1 Edoxaban: 1	Aspirin: 13	Aspirin and rivaroxaban: 1	
TPO-RA		6	3	1	5
	34	Acenocoumarol: 4 Apixaban: 1 Enoxaparin: 1	Aspirin: 3	Aspirin and Acenocumarol: 1	
Corticoids		1	1	1	0
	4	Enoxaparin: 1	Aspirin and clopidogrel: 1	Aspirin and bemiparin: 1	
IGIV	2				0
Combined Therapies	1 corticoids + RTX 1 corticoids + TPO-RA 1 corticoids + IGIV 2 corticosteroids + Azathioprine + TPO-RA 2 IGIV+TPO-RA				0

IGIV, intravenous immunoglobulin; RTX, rituximab; TPO-RA, thrombopoietin receptor agonists.

**Table 2:** Characteristics of the patients with immune thrombocytopaenia with and without suspected COVID-19.

		ITP with Possible COVID-19 *; *N* (%)	ITP Recognised as Non-COVID-19; *N* (%)	Chi-Squared, df, *p*
n		24	119	
ITP	Newly diagnosed Persistent Chronic	3 (12.5%) 0 (0.0%) 21 (87.5%)	2 (1.6%) 8 (6.7%) 109 (91.6%)	7.078; 1; 0.0078 1.709; 1; 0.191 0.406; 1; 0.524
Age (years)		59 ± 19	61 ± 19	
Therapeutic Treatment for ITP	No treatment Corticoids IGIV TPO-RA Combined therapy	18 (75.0%) 0 (0.0%) 0 (0.0%) 4 (16.7%) 2 (8.3%)	78 (65.6%) 4 (3.4%) 2 (1.7%) 30 (25.2%) 5 (4.2%)	0.156; 1; 0.693 0.830; 1; 0.362 0.409; 1; 0.522 0.804; 1; 0.370 0.732; 1; 0.392
Splenectomised		6 (25.0%)	21 (17.7%)	1.972; 1; 0.160
With antithrombotic therapy		7 (29.2%)	27 (22.7%)	0.034; 1; 0.855
With musculoskeletal pain		14 (58.3%)	44 (37.0%)	0.1871; 1; 0.665
Accompanied lockdown		22 (91.7%)	104 (87.4%)	0.348; 1; 0.555
Vulnerable population		10 (41.7%)	56 (47.1%)	0.234; 1; 0.629

* Probable or confirmed cases of coronavirus infection, as defined in the text. TPO-RA, thrombopoietin receptor agonists; IGIV, immune globulin intravenous. The chi-squared analysis is shown, and *p* < 0.05 was considered statistically significant.

## Data Availability

Data is contained within the article or supplementary material.

## Supplementary Materials

The following materials are available online at https://www.mdpi.com/1648-9144/57/3/219/s1, Questionnaire.
