# Supplementary material for: Impact of COVID-19 Pandemic on Patients with Immune Thrombocytopaenia
Source: Medicina (Kaunas). 2021 Mar 1;57(3):219. doi: 10.3390/medicina57030219 (PMC8002062; doi:10.3390/medicina57030219)
Supplement: Supplementary file 1 [file medicina-57-00219-s001.pdf]

<u>Supplementary file</u>

Annex 1. Questionnaire to patients and variables collected in the study.

1. Demographic characteristics

Age

Type of ITP (chronic, persistent, new diagnoses)

Type of therapeutic regimen forITP: Corticoids, IGIV, TPO-RA, Rituximab...

Comorbidities, associated with greater susceptibility to developing a more severe form of COVID-19, such as diabetes mellitus, high blood pressure, chronic lung disease, HIV. Others: cardiovascular pathologies, stroke, anticoagulant or antiplatelet treatment for prior comorbidities.

2. Clinical data suggestive of COVID-19 infection

Have you had any of the following symptoms in the last two months? fever >38°C, sore throat, persistent dry cough, shortness of breath, problems with smell or taste of food.

Have you had "atypical" muscle and/or joint pain (tingling, heat, cramps in areas without external stimuli, more tired than usual, loss of strength or sensation and/or weakness in walking?

Have any of the people who are under lockdown with you or in close contact with you if you are working had these symptoms?

If you've had these symptoms, have you contacted a healthcare professional?

Who did you contact?

Have you had any diagnostic tests (PCR)?

3. Managing COVID-19

Centre where the disease was managed

Communication with the HTC, if the patient went to another hospital

Hospital admission

Treatment for SARS-CoV-2 infection

4. Treatment for blood disorder

Have you continued with the same treatment and guidance prescribed by the hematologist? If it has not been followed up, please indicate the reason:

problems when collecting from the pharmacy, fear of being left without treatment,

fear of infection...

5. Assessment of pain and impact of lockdown on the locomotive system:

Have you had or do you have pain in your musculoskeletal system, related to your hemophilia?

If you have had pain or painful sensations: indicate location, intensity of pain from 0-10, type of pain. If you've been in pain: how have you managed it? Have you taken any medication?

Have you been able to adapt your regular physical activity to the lockdown? If yes, what activity have you done?

6. Psychosocial aspects:

Do you consider that you have been under a stricter lockdown than the rest of the population because of your ITP?

Are you spending your lockdown alone or with someone else?

Independence in carrying out the basic activities of daily life: Do you need help from other people for activities?

Who are you getting that help from?

During lockdown have you had to cope without people who usually help you with your daily activities?

Did you feel like you had support? From patient associations, hospital health staff, social services...

Do you have any degree of recognized disability/dependency?

Are you still working in the same job?

7. Aspects of quality of care:

Were you waiting for any appointments for screening, factor collection, rehabilitation, training, or surgery scheduling with the hospital for screening?

If you were, was it cancelled or postponed?

If it had been cancelled or postponed, was it by a call from the hospital or by your own decision?

With regard to factor collection at the Pharmacy Service, have you or any family member collected the treatment?

Did you have any trouble collecting the treatment?
